# CD4^+^ T Cells Promote IgG Production in MHC-Independent and ICAM-1-Dependent Manners in Pristane-Induced Lupus Mice

**DOI:** 10.1155/2022/9968847

**Published:** 2022-01-21

**Authors:** Shuai Liu, Yu-mei Li, Jin-zhi Li, Shu-jun Wang, Ping Ji, Mei-yu Zhang, Ying Wang

**Affiliations:** ^1^Shanghai Institute of Immunology, Department of Immunology and Microbiology, Shanghai Jiao Tong University School of Medicine, Shanghai 200025, China; ^2^Department of Blood Transfusion, Shanghai Ninth People Hospital, Shanghai Jiao Tong University School of Medicine, Shanghai 201999, China

## Abstract

Systemic lupus erythematosus (SLE) is an autoimmune disease characterized by autoantibody production and chronic inflammation. The etiology and pathogenesis of SLE are complicated in which dysfunction of CD4^+^ T cells is largely engaged. In this study, we investigated the manners of CD4^+^ T cells in antibody production in a lupus-like mouse model through peritoneal injection of pristane reagent. With the increase in total IgG/IgM and autoantibody production after 6 months, CD4^+^ T cells exhibited activated phenotypes with the elevated CD44, ICOS, OX40, and PD-1 expression. Pristane injection induced the increase in IgM levels in both wild-type and T cell-deficient TCR*α*^−/−^ mice whereas IgG, IgG1, and IgG2a production was impaired. When adoptively transferring CD4^+^ T cells into T cell-deficient mice or coculturing CD4^+^ T cells and B cells *in vitro*, it was found that CD4^+^ T cells derived from pristane-treated mice could help the production of total IgG as well as IgG1/IgG2a in a more efficient manner both *in vivo* and *in vitro*. While MHC was dispensable for IgG production, ICAM-1 likely functioned as an attenuating factor for IgG production. Our study thus reveals that CD4^+^ T cells in pristane-treated mice play important roles in IgG production, which implies the critical roles in the induction of pathological autoantibodies in MHC-independent and ICAM-1-dependent manners.

## 1. Introduction

Systemic lupus erythematosus (SLE) is a severe and heterogeneous systemic autoimmune disease. It is characterized by autoantibody (autoAb) production and immune complex (IC) deposition that mediate severe tissue destruction including the brain, blood, heart, and kidney [1–3]. The loss of B cell tolerance is one of the well-documented etiologies of SLE both in mice and in humans. Hyperactivation of B cells leads to abnormal generation of autoAbs to certain self-antigens including double-strand DNA and ribosome RNA [4]. AutoAbs circulate in the periphery and form autoAb-antigen immune complex (IC) depositing in terminal organs leading to tissue damages [5, 6]. Unlike IgM, autoAbs with IgG subtypes are more likely pathogenic. Introduction of IgGs specific to self-DNA into normal mice induces lupus-like glomerulonephritis [7, 8]. In contrast, injection of DNA-specific IgM rarely causes the autoimmune lesions in mice [9, 10]. These evidences support that IgG-type autoAbs are more crucial for the onset and progression of the disease [11, 12].

Multiple investigations from SLE patients and lupus-prone mice reveal that T cell-dependent antibody responses are the main drivers of SLE. There are two major pathways of pathological IgG production in extrafollicular foci (EF) and germinal center (GC). Pathogenic IgG is traditionally considered to be the result of aberrant GC responses. Once self-antigens (such as dsDNA) are exposed to antigen-presenting cells (APC), B cells are activated and migrate to the T-B border [13]. At the same time, CD4^+^ T cells are activated by dendritic cells (DCs) in the T cell zone, migrate to the follicular zone, and differentiate into follicular T helper cells (Tfh) characterized as ICOS^hi^PD-1^hi^CXCR5^+^ expression [14]. Subsequently, cognate T-B interactions drive the proliferation, some of which experience class switch recombination (CSR) and somatic hypermutation (SHM) [15–17]. Inducible T cell costimulator (ICOS) costimulation was reported to be involved in T-B interaction in GC [18]. ICOS costimulation enhances calcium fluxes in Tfh and drives rapid externalization of CD40L from intracellular vesicles [18, 19]. GC B cells upregulate the ICOS ligand (ICOSL) upon CD40-CD40L ligation [18]. Therefore, ICOS and CD40 coordinately constitute the intracellular positive feedback between cognate T-B interaction in GC, facilitating the differentiation of high-affinity IgG-secreting B cells and memory B cells [18]. It is not out-of-expectation that abnormal activation of CD4^+^ T cells might be crucial for the production of pathogenic antibodies. In fact, CD4^+^ T cells have been shown to play detrimental roles in many autoimmune diseases, including SLE, Sjögren's syndrome, Hashimoto's thyroiditis, and systemic sclerosis [20, 21]. They not only produce multiple effector cytokines for pathological inflammation but also provide extra signals for B cell activation, which might in turn interrupt the self-tolerance of B cells. Generation of pathogenic autoAbs in SLE, to some extent, reflects T cell-mediated extra help for B cell differentiation [22–25]. Although many studies reveal the roles of CD4^+^ T cells in the development and pathogenesis of autoimmune diseases, its mechanisms remain to be elucidated during pathological processes.

In this study, we intended to clarify the interaction patterns of CD4^+^ T cells with B cells in promoting IgG production under the pathogenesis of lupus. We, therefore, utilized a pristane-induced lupus mouse model to dissect whether activated CD4^+^ T cells from pristine-treatment mice promoted B cell activation and differentiation in the MHC and ICAM-1-dependent manners. While MHC is a key molecule to be engaged in antigen recognition, ICAM-1 is more inclined to mediate CD4^+^ T-B adhesion at the first step of T-B interaction. Our study will thus provide direct evidence of how CD4^+^ T cells interacting with B cells to participate in the pathogenesis of pristine-induced lupus in the mice, which may imply new strategies for SLE treatment.

## 2. Materials and Methods

### 2.1. Mice

TCR*α*^−/−^ mice (on a C57BL/6 background) were purchased from the Model Animal Research Center of Nanjing University (Nanjing, China) and maintained in the Department of Animal Science of Shanghai Jiao Tong University School of Medicine under the specific-pathogen-free (SPF) conditions. BALB/c, C57BL/6 mice, and TCR*α*^−/−^ mice at 6~8 weeks old were injected intraperitoneally (i.p.) with a single dose of 0.5 mL pristane (Sigma-Aldrich, St. Louis, MO, USA) or 0.5 mL sterile phosphate buffer saline (PBS). Peripheral whole blood of 200 *μ*L was collected monthly from the eye veins after the injection for the preparation of serum. Mice were sacrificed at 6 months for further experiments. All animal experiments were performed in compliance with the animal care and used the guidelines certificated by Shanghai Jiao Tong University School of Medicine. Unless stated, 3-5 female mice were used in each experiment.

### 2.2. Enzyme-Linked Immunosorbent Assay (ELISA)

For determination of total IgG/IgG1/IgG2a/IgM, plates (Corning, Corning, NY, USA) were coated with 2 *μ*g/mL goat anti-mouse IgG (H+L) or 4 *μ*g/mL goat anti-mouse IgM (SouthernBiotech, Birmingham, AL, USA) diluted in bicarbonate/carbonate coating buffer (15 mM Na_2_CO_3_ and 35 mM NaHCO_3_, pH 9.6) and incubated at 4°C overnight. To determine autoAbs, dsDNA (0.3 *μ*g/mL) and Sn-RNP (0.3 *μ*g/mL) were coated in the plates and incubated overnight. After washing 3 times with PBST (PBS containing 0.05% Tween-20) (Sangon Biotech, Shanghai, China), the wells were blocked with 200 *μ*L/well PBS containing 5% skimmed milk powder at room temperature (RT) for 2 h. Mouse serum or coculture supernatants were serially diluted in PBST containing 3% skimmed milk powder. 100 *μ*L diluted samples were added to the wells, and the plates were incubated at RT for 2 h. After washing 3 times with PBST, the wells were incubated with horseradish peroxidase- (HRP-) conjugated goat anti-mouse IgG (H+L) (for total IgG, anti-dsDNA, and anti-Sn-RNP), HRP-conjugated goat anti-mouse IgM (SouthernBiotech), HRP-conjugated goat anti-mouse IgG1 (SouthernBiotech), or alkaline phosphatase- (AP-) conjugated goat anti-mouse IgG2a Abs (Rockland, Gilbertsville, PA, USA), respectively, at RT for 2 h. Tetramethylbenzidine (TMB) (BD Biosciences, San Diego, CA, USA) solution (for HRP) or VISIGLO AP CHEMILUM SUBSTRATE (Sigma) solution (for AP) (100 *μ*L/well) was added. The plates were incubated at RT in the dark for 15 min. The reaction was stopped by adding 1 M H_2_SO_4_ (50 *μ*L/well) solution (for HRP) or 5 M NaOH (50 *μ*L/well) solution (for AP). The absorbance at 450 nm (for TMB) or 405 nm (for VISIGLO AP CHEMILUM SUBSTRATE) was detected within 5 min by using a PowerWaveXS2 microplate spectrophotometer (BioTek, Burlington, VT, USA).

### 2.3. Flow Cytometry

After six months of i.p. injection of the pristane, mice were sacrificed and the splenocytes were isolated from the spleens by density gradient centrifugation using LymphoprepT^M^ reagent (Axis-shield, Oslo, Norway). Cells were stained by fluorescence-conjugated antibodies (Abs) including APC-anti-mouse CD3, FITC-anti-mouse CD4, APC-anti-mouse OX40, PE-anti-mouse ICOS, AF700-anti-mouse CD38 (from eBioscience, Waltham, MA, USA), FITC-anti-mouse CD44, PE-anti-mouse CD62L, APC-anti-mouse ICAM-1, and FITC-anti-mouse IgG1 (from BD Biosciences). Cells were incubated at 4°C for 30 min and acquired on an LSR Fortessa (BD Biosciences). For intracellular staining, cells were fixed and permeabilized using the Transcription Factor Buffer Set (BD, Biosciences) according to the manufacture's protocol. Cells were incubated with PE-Cy7-anti-mouse Foxp3 for 40 min. Cells were acquired using an LSRFortessa flow cytometer (BD, Biosciences). Data analysis was carried out with FlowJo 7.6 software (Tree Star, Ashland, OR, USA).

### 2.4. Immunofluorescence Assay (IFA)

Frozen sections of the kidneys were subjected to an immunofluorescence assay (IFA) by using one-step staining for IgG deposition. FITC-labeled anti-mouse IgG Ab (Abcam, #ab6785) was used in the assay. Sections were mounted with Fluoromount-G reagent (YEASEN Biotech, Shanghai, China) before the observation. Fluorescence signal was determined under a TCS SP8 confocal microscope (Leica, Solms, Germany).

### 2.5. Adoptive Transfer

CD4^+^ T cells were prepared from the splenocytes by using a CD4^+^ T cell negative isolation kit (Miltenyi Biotec, Bergisch Gladbach, Germany) according to the manufacturer's instructions. The purity was determined by flow cytometry. 1 × 10^5^ CD4^+^ T cells from PBS or pristane-treated C57BL/6 mice were intravenously (i.v.) injected into TCR*α*^−/−^ mice (on a C57BL/6 background). Fourteen days after adoptive transfer, mice were sacrificed and the sera were collected. IgM, IgG, and IgG subclasses including IgG1 and IgG2a were quantified by ELISA.

### 2.6. *In Vitro* T-B Cell Coculture

3 × 10^4^ purified CD4^+^ T cells from PBS or pristane-treated BALB/c mice were cultured with 9 × 10^4^ CD4^−^ splenocytes from wild-type BALB/c mice or the splenocytes from TCR*α*^−/−^ mice (on a C57BL/6 background) in 200 *μ*L RPMI1640 medium (Gibco, Waltham, MA, USA) containing 10% fetal calf serum (FCS) (Gemini, Woodland, California, USA), 10 mM HEPES buffer (Gibco), 200 *μ*g/mL gentamicin (Gibco), and 50 *μ*M *β*-mercaptoethanol (Gibco). IgM and IgG levels were quantified in culture supernatants with 1 : 2 dilution at different time points (days 2, 4, 6, 8, and 12) by ELISA. In blockade experiment, anti-ICAM-1 and isotype Abs (5 *μ*g/mL) were added during the *in vitro* coculture for 8 days.

### 2.7. Statistical Analysis

Data were presented as means ± standard error of means (S.E.M). Statistical analyses were performed by using GraphPad Prism 5.0 software (GraphPad Prism, San Diego, CA, USA). Statistic difference was determined by the unpaired Student *t*-test for the data with Gaussian distribution and by the Mann-Whitney test for those with non-Gaussian distribution. Unless stated, *p* < 0.05 was considered statistically significant.

## 3. Results

### 3.1. Hyperglobulinemia in Pristane-Induced Lupus-Like Mice

**Figure 1:**
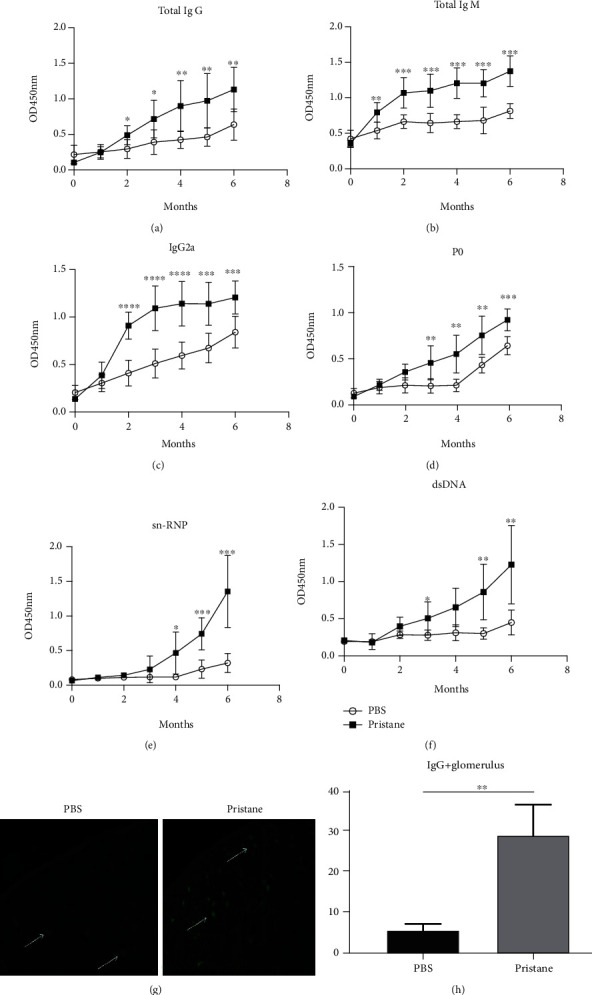
Serum immunoglobulin levels in pristane-induced lupus-like mice. Sera were collected before the injection and at 6 months post-PBS or pristane treatment. The levels of total IgG (a), total IgM (b), IgG2a (c), and anti-P0 (d), anti-SnRNP (e), and anti-dsDNA (f) autoantibodies in the serum were determined by an ELISA assay. Furthermore, the kidneys were collected when the mice were sacrificed at 6 months after PBS/pristane injection. The deposition of immune complexes (g) in the kidney was detected by the immunofluorescence assay. (h) Statistic analysis of immune complexes in the kidneys. Data were pooled from three independent experiments with 5 mice per group in each experiment. ^∗^*p* < 0.05; ^∗∗^*p* < 0.01; ^∗∗∗^*p* < 0.001; ^∗∗∗∗^*p* < 0.0001.

Increase in serum autoAbs levels is one of the key manifestations of SLE. One dose injection of pristane largely recapitulates the pathology of SLE in BALB/c mice. Serum Ig, including total IgM, IgG, IgG1, and IgG2a levels in pristane-induced mice, were detected at 6 months by an ELISA assay. It was shown that total IgM and IgG levels of pristane-treated mice were higher than those in PBS-treated control mice at 6 months (Figures 1(a) and 1(b)). IgG2a autoAb is considered to be the most pathogenic, whereas IgG1 displays the poorest pathogenicity in the mice [26]. In our study, we found that IgG2a level in pristane-treated mice increased significantly at 6 months after the injection (Figure 1(c)). More specifically, autoAbs to P0, dsDNA, and SnRNP were elevated in the sera of pristane-treated mice when compared to control mice (Figures 1(d)–1(f)) with more deposition of ICs in the kidneys consequently (Figures 1(g) and 1(h)). These results confirm the presence of hyperglobulinemia in pristane-treated mice, which is associated with lupus-like pathogenesis.

### 3.2. Increased Expression of Activated Markers on CD4^+^ T Cells and B Cells in the Spleens of Pristane-Induced Lupus-Like Mice

**Figure 2:**
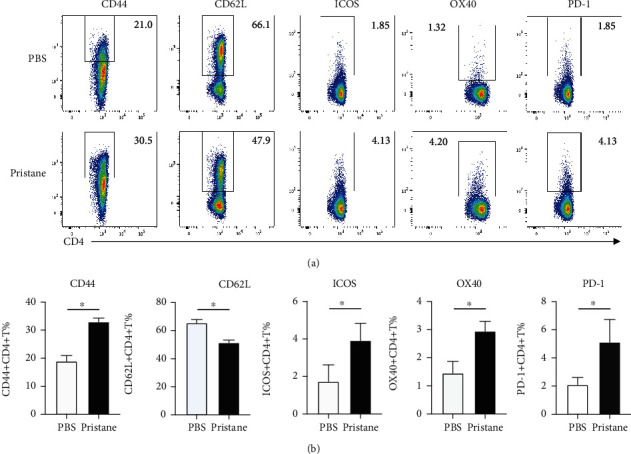
Activated phenotypes of CD4^+^ T cells in the pristane-induced lupus-like mouse model. The splenocytes were collected at 6 months after i.p. injection of PBS or pristane. (a) The expression levels of CD62L, CD44, ICOS, OX40, and PD-1 on CD4^+^ T cells. (b) Statistic percentages of CD62L, CD44, ICOS, OX40, and PD-1 on CD4^+^ T cells in the spleens of PBS or pristane-treated mice. ^∗^*p* < 0.05.

Antibody-producing cells (also called as plasma cells) can arise through either direct differentiation in EF or GCs with the help of CD4^+^ T cell [27]. There exist increased numbers of circulating pre-GC B cells, CCR7^lo^PD-1^hi^CXCR5^+^CD4^+^ T cells, and memory B cells in SLE patients as well as in lupus-like mice, indicating the existence of CD4^+^ T cell activation and subsequently enhanced GC responses [28–30]. This is associated with elevated generation of plasma cells within GCs and contributes to the development of lupus pathogenesis in both mice and humans [27]. Therefore, we tested the phenotypes of CD4^+^ T cells and B cells derived from pristane and PBS-treated mice by flow cytometry. It was apparent that the expression of CD44 increased significantly whereas that of CD62L decreased significantly on CD4^+^ T cells from the pristane-treated group when compared to the PBS group (Figures 2(a) and 2(b)). More interestingly, costimulatory molecules, which are important for T-B interaction including ICOS, OX40, and PD-1, were also elevated on CD4^+^ T cells from pristane-treated mice (Figures 2(a) and 2(b)). These results indicate that CD4^+^ T cells derived from pristane-treated mice display activated phenotypes, which might facilitate B cell activation and differentiation.

### 3.3. CD4^+^ T Cells Are More Inclined to Promote IgG Production in Pristane-Induced Lupus-Like Mice

**Figure 3:**
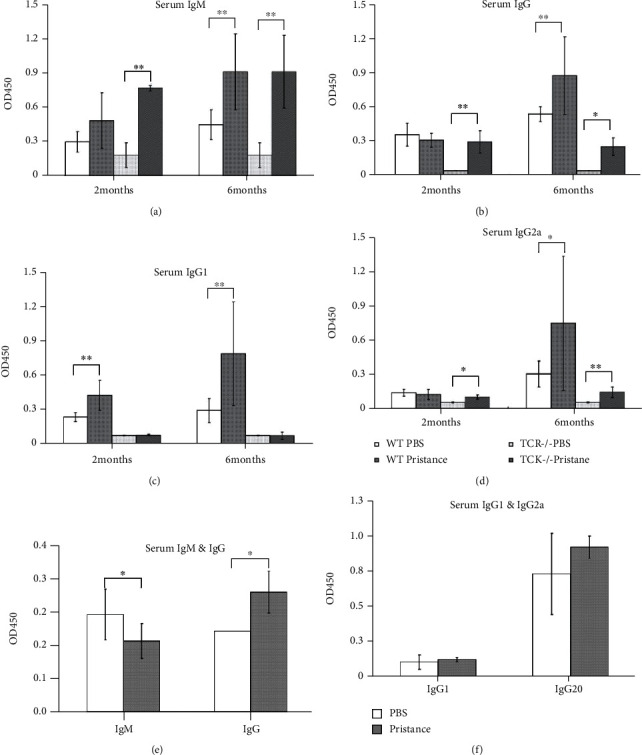
CD4^+^ T cells are more inclined to promote IgG production in pristane-induced lupus-like mice. Serum from PBS or pristane-treated C57BL/6 and TCR*α*^−/−^ mice (on a C57BL/6 background) were subjected to the determination of total IgM/IgG and IgG subtype IgG1/IgG2a by the ELISA assay. OD_450_ value was detected to represent IgM (a), IgG (b), IgG1 (c), and IgG2a (d) levels in the serum of the mice. CD4^+^ T cells sorted from PBS and pristane-treated C57BL/6 mice were i.v. injected into TCR*α*^−/−^ mice (1 × 10^5^ cells/body), respectively, and the serum of TCR*α*^−/−^ mice were collected 14 days after CD4^+^ T cells were transferred. The levels of total IgM/IgG (e) and IgG subtype IgG1/IgG2a (f) in the serum were determined by ELISA. Each group had at least six mice for the analysis. ^∗^*p* < 0.05; ^∗∗^*p* < 0.01.

To further investigate the potential roles of CD4^+^ T cells in elevated production of Igs upon pristane injection, age- and gender-matched wild-type (WT) and TCR*α*^−/−^ C57BL/6 background mice were administered with one dose of pristane to induce lupus-like phenotypes while PBS-treated mice as controls. After 6 months, Igs production was compared between WT and TCR*α*^−/−^ mice. The levels of serum IgM in the pristane-treated group at 2 months and 6 months were significantly higher than those in the PBS-treated group, but comparable between WT and TCR*α*^−/−^ mice (Figure 3(a)). However, at 6 months after pristane injection, total IgG level was dramatically lower in TCR*α*^−/−^ mice than that in WT mice whereas at 2 months, it was comparable between two groups (Figure 3(b)). When comparing IgG1 and IgG2a levels between WT and TCR*α*^−/−^ mice, it was obvious that both IgG1 and IgG2a were lower in TCR*α*^−/−^ mice than those in WT counterparts both at 2 months and 6 months (Figures 3(c) and 3(d)). The results above indicated that IgG production was impaired with the deficiency of T cells in TCR*α*^−/−^ mice.

Furthermore, purified CD4^+^ T cells were isolated from pristane or PBS-treated C57BL/6 mice and adoptively transferred to TCR*α*^−/−^ mice. 14 days later, serum IgM, IgG, IgG1, and IgG2a levels were determined by ELISA. It was revealed that IgM level was slightly decreased whereas IgG level was dramatically increased in TCR*α*^−/−^ mice receiving CD4^+^ T cells from pristane-treated mice (Figure 3(e)). Simultaneously, a slight increase in IgG2a levels was observed as well in TCR*α*^−/−^ mice with the transfer of CD4^+^ T cells derived from pristane-treated mice when compared to the PBS group (Figure 3(f)). These results, therefore, indicate that CD4^+^ T cells play important roles in the induction of IgG production in pristane-induced lupus-like mice.

### 3.4. Activated CD4^+^ T Cells Promote IgG Production in an MHC-Independent Way

**Figure 4:**
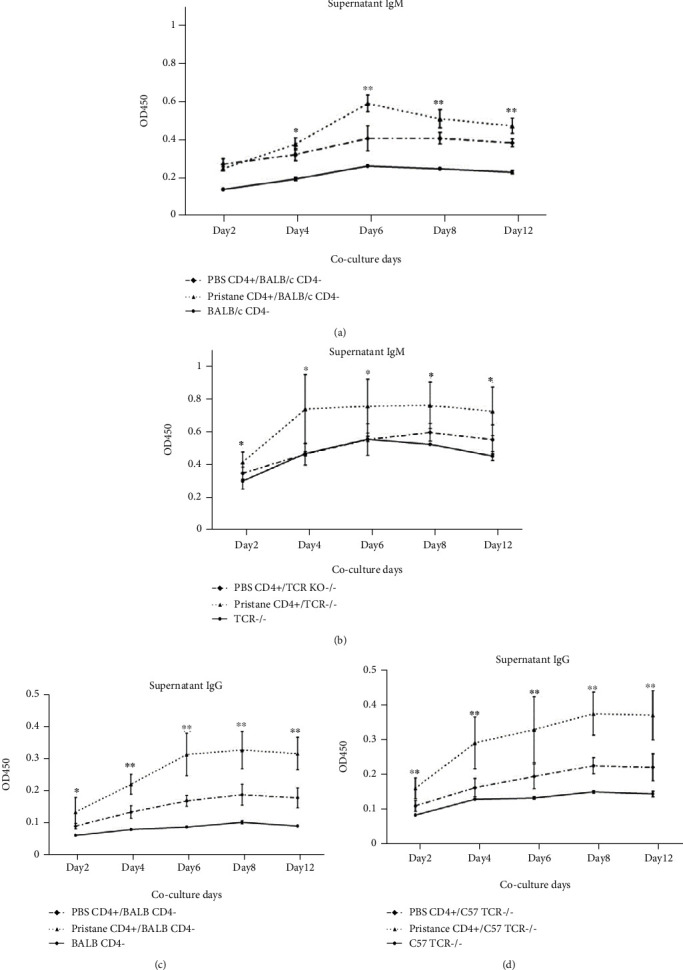
Activated CD4^+^ T cells can promote IgG production in an MHC-independent way. CD4^+^ T cells were sorted from PBS and pristane-treated BALB/c mouse (6 months later) by MACS. 3 × 10^4^ CD4^+^ T cells were cultured with 9 × 10^4^ CD4^−^ splenocytes derived from wild-type BALB/c or TCR*α*^−/−^ mice (on a C57BL/6 background). Total IgM (a, b) and IgG (c, d) levels in the supernatants at different time points (days 2, 4, 6, 8, and 12) were quantified by ELISA. Each group had at least six mice for the analysis. ^∗^*p* < 0.05; ^∗∗^*p* < 0.01.

Several studies introduced the involvement of costimulatory molecules on activated CD4^+^ T cells, such as PD-1 [31] and ICOS [32, 33] in T-B interaction and B cell differentiation in GC responses. However, whether MHC is also functional in this process is not well addressed. Therefore, CD4^+^ T cells were sorted from pristane and PBS-treated BALB/c mice and cocultured with CD4^−^ splenocytes from BABL/c mice or the splenocytes from TCR*α*^−/−^ mice with C57BL/6 genetic background. As shown in Figures 4(a) and 4(c), IgM and IgG levels in the supernatants derived from pristane-treated derived CD4^+^ T cells coculturing with CD4^−^ responder cells from BALB/c mice were higher than those from PBS-treated mice derived CD4^+^ T cell coculture system, which is consistent with the results from adoptive transfer. Interestingly, similar results were observed when CD4^+^ T cells derived from pristane-treated mice were cocultured with the splenocytes from TCR*α*^−/−^ mice with increased IgM and IgG in the culture supernatants as well (Figures 4(b) and 4(d)). Therefore, activated CD4^+^ T cells from pristane-treated mice likely promote B cells to generate IgM and IgG antibodies in a noncognate MHC-independent way.

### 3.5. ICAM-1 Blockade Facilitates IgG Production during the Interaction between Activated CD4^+^ T Cells and B Cells

**Figure 5:**
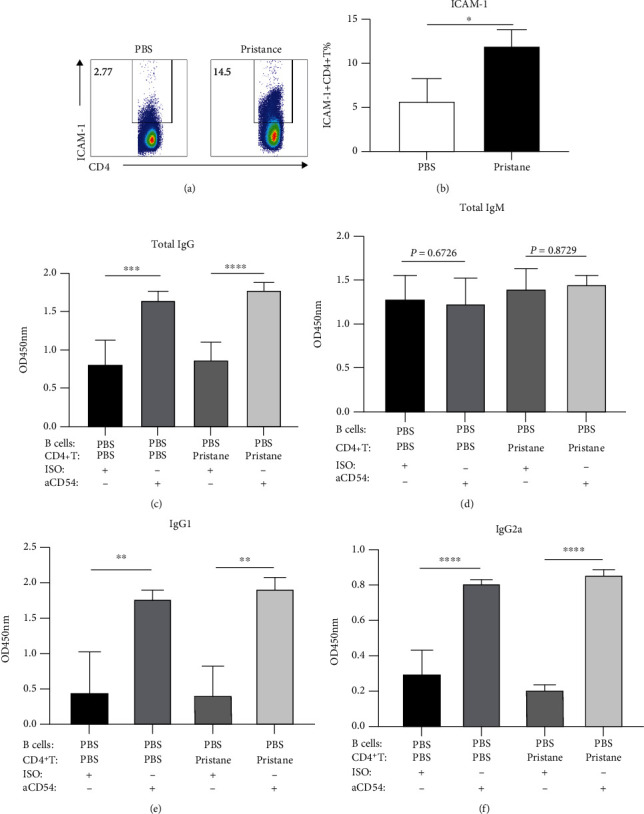
ICAM-1 attenuates IgG production in the coculture of CD4^+^ T cell and B cell interaction from PBS or pristane-treated mice. CD4^+^ T cells and B cells were sorted from PBS and pristane-treated BALB/c mouse (6 months later) by MACS, respectively. 3 × 10^4^ CD4^+^ T cells were cultured with 9 × 10^4^ B cells derived from PBS or pristane mice (on a BALB/c background). (a) Flow cytometric analysis of ICAM-1 expression on CD4^+^ T cells. (b) Increased expression levels of ICAM-1 on CD4^+^ T cells from lupus-like mice. (c–f) Total IgG (c), IgM (d), IgG1 (e), and IgG2a (f) levels in the supernatants of different coculture groups (T+B, T+B+ISO, and T+B+anti-ICAM-1) were quantified by the ELISA assays. Each group had at least five mice for the analysis. Data are represented by mean of OD_450_ values with bar of SEM from five mice. ^∗^*p* < 0.05; ^∗∗^*p* < 0.01; ^∗∗∗^*p* < 0.001; ^∗∗∗∗^*p* < 0.0001.

ICAM-1 is a key adhesion molecule that is involved in the interaction between antigen-presenting cells and T cells for T cell activation [34]. Several studies also reported the roles of ICAM-1 in B cell differentiation in GC, such as prevention of apoptosis of B cells [35, 36] and interaction between B cells and follicular helper T cells. By using *in vitro* culture systems, we intended to investigate the effects of ICAM-1 on IgM and IgG production by B cells from pristane-treated mice. Interestingly, with the significant increase of ICAM-1 expression on CD4^+^ T cells (Figures 5(a) and 5(b)), we found that IgG, IgG1, and IgG2a levels were increased dramatically in the supernatants of CD4^+^ T cell and B cell coculture with anti-ICAM-1 Ab when compared to isotype Ab no matter whether CD4^+^ T cells and B cells came from PBS or pristane-treated mice (Figures 5(c), 5(e), and 5(f)). However, ICAM-1 blockade had no effect on IgM content in the coculture supernatant (Figure 5(d)). These data strongly suggested that ICAM-1 might be an attenuated factor of IgG production during CD4^+^ T cell and B cell interaction.

**Figure 6:**
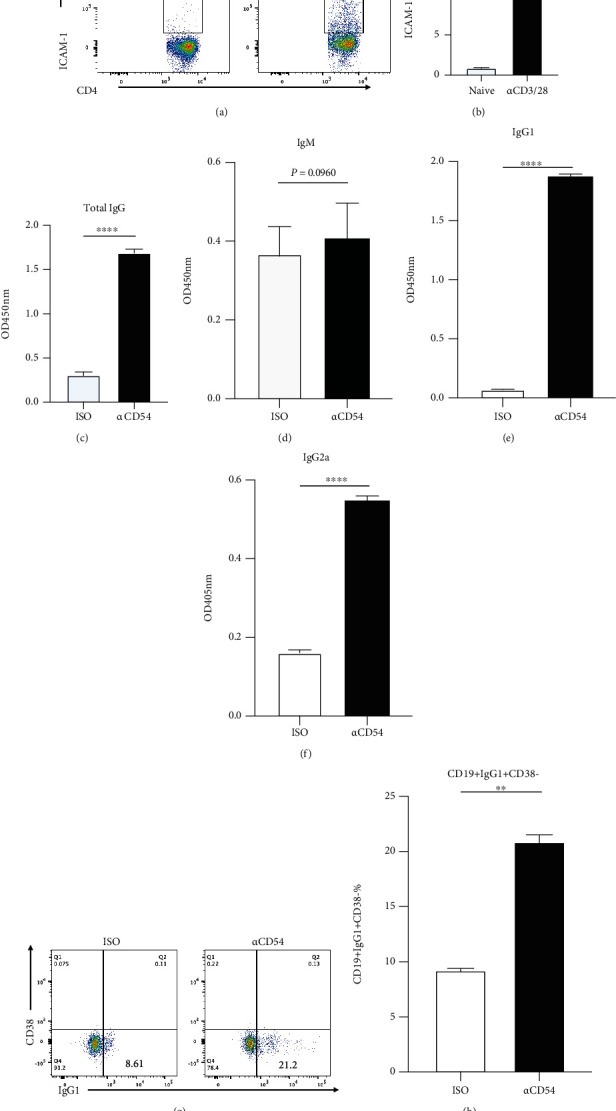
ICAM-1 attenuates IgG production in the coculture of *in vitro* activated CD4^+^ T cell and B cells. Wildtype CD4^+^ T cells and B cells were sorted from 6- to 8-week-old BALB/c mice by MACS, respectively. CD4^+^ T cells were stimulated by anti-CD3 and anti-CD28 antibodies for 72 hours. Then, 3 × 10^4^ activated CD4^+^ T cells were cultured with 9 × 10^4^ B cells. (a) Flow cytometric analysis of ICAM-1 expression on CD4^+^ T cells. (b) Increased expression levels of ICAM-1 on *in vitro* activated CD4^+^ T cells. (c–f) Total IgG (c), IgM (d), IgG1 (e), and IgG2a (f) levels in the supernatant with or without anti-ICAM-1 blocking antibodies were determined by ELISA. (g, h) Determination of differentiated B cells after the coculture. Each group had at least five mice for the analysis. ^∗∗^*p* < 0.01; ^∗∗∗∗^*p* < 0.0001.

Pristane-induced lupus-like mice were sacrificed at least 6 months after the injection. To exclude the effects of aging-related activation on the IgG production in the coculture system, we harvested the spleen from the 6~8 weeks BALB/c mice and cocultured B cells with CD4^+^ T cells which were stimulated by anti-CD3/28 antibody for 72 hours. Firstly, we confirmed that ICAM-1 expression was significantly upregulated upon CD4^+^ T cells after the stimulation (Figures 6(a) and 6(b)). As expected, ICAM-1 blockade had a slight effect on IgM content (Figure 6(d)) but dramatically facilitated IgG, IgG1, and IgG2a production in the coculture supernatant (Figures 6(c), 6(e), and 6(f)). Consistent with the IgG level, more class-switched B cells (indicated by CD19^+^IgG1^+^CD38^−^ B cells) were detected in the coculture system with anti-ICAM-1 Ab when compared to isotype Ab (Figures 6(g) and 6(h)). These data reveal that ICAM-1 plays a negative role in IgG production during the interaction between activated CD4^+^ T cells and B cells.

### 3.6. ICAM-1 Blockade Leads to the Decrease in Treg in the Coculture of CD4^+^ T Cells and B Cells

**Figure 7:**
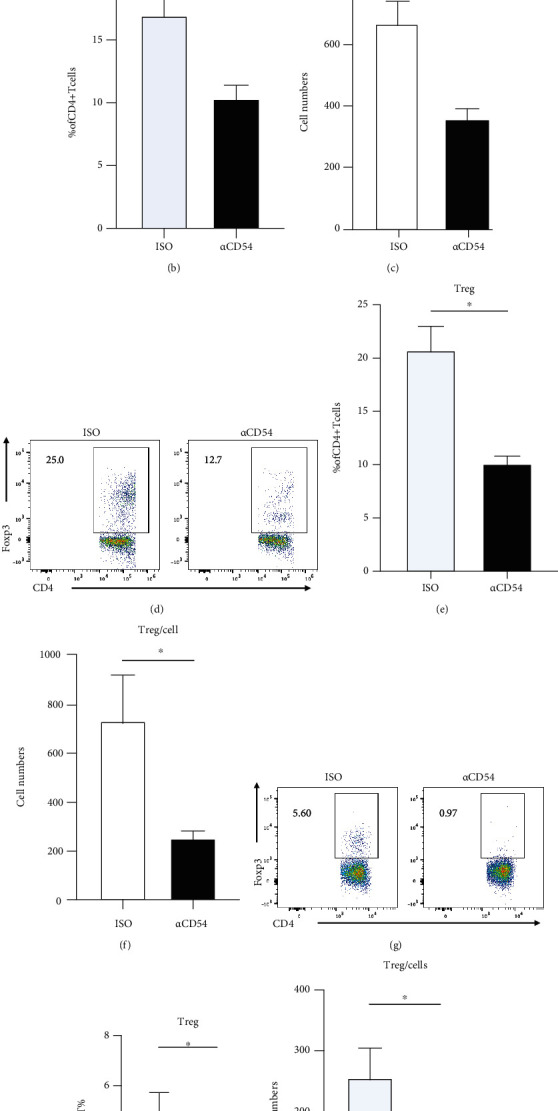
ICAM-1 blockade leads to the decrease in Treg in the coculture of CD4^+^ T cells and B cells. CD4^+^ T cells and B cells were sorted from PBS and pristane-treated BALB/c mouse (6 months later) by MACS, respectively. 3 × 10^4^ CD4^+^ T cells originated from PBS or pristane-treated mice were cultured with 9 × 10^4^ B cells derived from PBS mice (on a BALB/c background). After coculturing for 8 days, Foxp3^+^CD4^+^ Treg cells were determined by flow cytometer in the coculture of CD4^+^ T cells and B cells from the PBS group (a–c) and that from the pristane-treated group (d–f). (g–i) The proportions and numbers of Treg in activated CD4^+^ T-B coculture upon ICAM-1 blockade were determined by flow cytometer. ^∗^*p* < 0.05; ^∗∗^*p* < 0.01.

Previous studies suggested that regulatory T cells (Treg) could inhibit antibody secretion, CSR, SHM through metabolic reprogramming, and epigenetic remodeling of B cells. We have observed that the addition of anti-ICAM-1 antibodies led to the increased IgG production in the coculture systems of PBS or pristane-treated CD4^+^ T cells and B cells. We, therefore, detected the percentages of Treg after the coculture. It was found that the percentages and the numbers of Treg were both dramatically decreased upon ICAM-1 blockade no matter CD4^+^ T cells were derived from PBS-treated mice (Figures 7(a)–7(c)) or pristane-treated mice (Figures 7(d)–7(f)). Furthermore, we also observed that the proportions and numbers of Treg were both strongly decreased upon ICAM-1 blockade during the interaction between activated CD4^+^ T and B cells (Figures 7(g)–7(i)). Considering the regulations of Treg on B cell differentiation and antibody production, our data imply that ICAM-1 blockade-mediated IgG increase might be due to the suppression of Treg cells in the CD4^+^ T-B coculture.

## 4. Discussion

In SLE, abnormal B cell activation and autoAb productions are demonstrated to trigger the formation and deposition of immune complexes (ICs) and multiplex tissue injuries largely due to excessive CD4^+^ T cell help and the loss of immune tolerance [37]. In this study, we have investigated the exact roles and relevant mechanisms of CD4^+^ T cells in abnormal IgG production using a pristane-induced lupus-like mouse model. As reported previously, we observed that total IgG, IgG1, and IgG2a levels as well as autoantigen-specific Abs were elevated in pristane-treated mice when compared to those with PBS treatment. This is consistent with the accumulating evidence that hyperreactive B cells play a paramount role in the pathogenesis of SLE with the production of autoAbs via plasma cells [38, 39] while autoAbs with IgG subtypes are more likely pathogenic according to previous studies [8, 11].

Production of total IgG and autoAbs in SLE is programmed by multiple factors [40, 41], among which IgG rather than IgM is undoubtedly crucial for the onset and progression of the disease. High-affinity IgG autoAbs are thought to arise through GC response including SHM and CSR, which are two crucial events in humoral immune response. How CD4^+^ T cells are involved in IgG production under SLE pathogenesis is not well addressed. Herein, we have demonstrated the critical roles of CD4^+^ T cell in helping the generation of IgG. Firstly, IgG levels in TCR*α*^−/−^ mice upon pristane treatment decreased significantly when compared with WT mice, but IgM levels were maintained in the mice with (WT) or without (TCR*α*^−/−^) T cells (Figure 3). Secondly, when adoptively transferring CD4^+^ T cells derived PBS or pristane-treated mice into TCR*α*^−/−^ mice, we found that CD4^+^ T cells from pristane-treated mice could facilitate the production of total IgG as well as IgG1/IgG2a in a more efficient manner whereas IgM levels were decreased in T cell-deficient mice. Thirdly, consistent with *in vivo* results, our *in vitro* study showed that CD4^+^ T cells from pristane-treated mice promoted the production of IgG more efficiently than CD4^+^ T cells from control mice as well (Figure 4(c)). Collectively, these emerging data indicate that CD4^+^ T cells in pristane-treated mice with activation markers can promote IgG production, which is critical in the induction of pathological autoantibodies in pristane-induced lupus mice.

Recent insights have implicated that CSR-oriented IgG production during certain infections does not require cognate T-B interactions [42]. Other studies indicate that T-independent CSR responses contribute to lupus occurrence in mice [43, 44]. Controversially, there are also studies in lupus-prone mice and SLE patients suggesting that T-dependent responses are the main driver of the disease [45, 46]. MHC molecules are the key factor in the initiation stages of thymus-dependent (TD) humoral immune response [47]. Considering that once CD4^+^ T cells are overactivated especially under pathogenic circumstances with intensive expression of costimulatory molecules on the surface and the production of cytokines, whether MHC is strictly required is not clear. Our results from *in vitro* coculture of MHC unmatched CD4^+^ T and B cells showed that CD4^+^ T cells from pristane-induced mice could induce IgM to convert into IgG similar to the coculture with MHC-matched CD4^+^ T cells and B cells. In fact, antibody production upon CD4^+^ T cell and B cell interaction in a MHC-independent way has been reported previously in *LAT^Y136F^* mice [48]. The intrinsic reasons between two models might be quite similar. One is that certain B cells are activated which is sufficient for further differentiation. Another is due to fact that the upregulation of multiple costimulatory molecules on pathogenic CD4^+^ T cells, including ICOS, PD-1, and OX40, probably provides strong connections between T cells and B cells to trigger CSR and terminal IgG production.

Unexpectedly, ICAM-1 seems to play contradictory roles in IgG production according to our *in vitro* coculture results. ICAM-1 is one of the adhesion molecules mediating adhesion between APC and T cells in the early stage of T cell activation. Its interaction with LFA-1 on T cells facilitates further cognate recognition of peptide-MHC on APCs and specific TCR molecules on T cells [49]. The investigation on the roles of ICAM-1 in humoral immunity has not been well addressed. Koopman et al. has reported the involvement of ICAM-1 in the prevention of apoptosis of B cells in the germinal center [36], while Zaretsky et al. provided the evidence on the supportive role of ICAMs for clonal selection [50]. However, no report described its roles in pathogenic IgG production. In this study, upon ICAM-1 blockade in *in vitro* CD4^+^ T and B cell culture system, IgG levels increased dramatically whereas IgM decreased on the contrary (data not shown), which suggests that the interaction between ICAM-1 and its ligand between CD4^+^ T cells and B cells attenuates IgM-to-IgG transition. This might reflect the alternative roles of ICAM-1 at the different stage of B cell terminal differentiation, which needs to be further investigated.

Treg cells have immunosuppressive function and play an important role in the induction and maintenance of self-tolerance. The reducing number and function of Tregs are also involved in the occurrence and development of organ inflammation in SLE [51, 52]. Scalapino and Daikh have reported that Treg cells injected into the lupus mice can control the inflammation injury and organ damage [53]. Most recently, Li et al. provided a gene-editing-based approach to convert defective Tregs into SuperTreg which can control inflammation and avoid the tissue injury in autoimmunity [54]. ICAM-1 is thought to participate in the formation of T-APC synapse, thus resulting in the T cell activation and differentiation. However, whether ICAM-1 influences the Treg differentiation is still unclear. In our study, ICAM-1 blockade *in vitro* can significantly reduce the Treg populations when total CD4^+^ T cells either from PBS or pristane-treated mice were cocultured with B cells. This might be the cellular mechanism of increased IgG content in the T-B coculture system upon ICAM-1 blockade. How ICAM-1 controls or influences Treg survival warrants further exploration.

## 5. Conclusion

In summary, our study provides the direct evidence on the involvement of pathogenic CD4^+^ T cells in IgG production in pristane-induced lupus-like mice. While MHC is not the key factor to mediated IgG production, ICAM-1 likely attenuates IgG production to some extent. Considering pristane-induced lupus-like mouse model recapitulates most of the key immunologic and clinical features of human SLE, the role of pathogenic CD4^+^ T cell in the generation of pathogenic IgG in human SLE might be similar. Once the underlying mechanisms are clarified, novel targets might be developed for new diagnosis and therapy to control SLE.

## Data Availability

All data generated or analyzed during this study are included in this article.

## Abbreviations

SLE: Systemic lupus erythematosus
MHC: Major histocompatibility complex
ICOS: Inducible T-cell costimulator
ICAM-1: Intercellular adhesion molecule 1
IC: Immune complex
CSR: Class switch recombination
GC: Germinal center
EF: Extrafollicular foci
TCR: T cell receptor
APC: Antigen-presenting cell
SHM: Somatic hypermutation
DC: Dendritic cell
Treg: Regulatory T cell.
